# Hypoxia matters: comparison of external and internal training load markers during an 8-week resistance training program in normoxia, normobaric hypoxia and hypobaric hypoxia

**DOI:** 10.1007/s00421-024-05442-1

**Published:** 2024-03-06

**Authors:** Lara Rodríguez-Zamora, Cristina Benavente, Irene Petrer, Paulino Padial, Rafa Timón, Javier Arguelles, Belén Feriche

**Affiliations:** 1 School of Health and Medical Sciences, Division of Sport Sciences, Örebro University, Örebro, Sweden; 2 Department of Physical Education and Sport, Faculty of Sport Sciences, University of Granada, Granada, Spain; 3 Faculty of Sport Sciences, University of Extremadura, Cáceres, Spain; 4 High Performance Center of Sierra Nevada, Spanish Sport Council, Granada, Spain

**Keywords:** Altitude, Hypoxic training, Monitoring load, Performance, Resistance training

## Abstract

**Purpose:**

To compare external and internal training load markers during resistance training (R_T_) in normoxia (N), intermittent hypobaric hypoxia (HH), and intermittent normobaric hypoxia (NH).

**Methods:**

Thirty-three volunteers were assigned an 8-week R_T_ program in either N (690 m, *n* = 10), HH (2320 m, *n* = 10), or NH (inspired fraction of oxygen = 15.9%; ~ 2320 m, *n* = 13). The R_T_ program (3x/week) consisted of six exercises, with three sets of six to 12 repetitions at ~ 70% of one repetition maximum (1RM) with the first session of each week used for analysis. 1RM in back squat and bench press was used to evaluate muscle strength before and after the program. External load was assessed by the volume load relative to body mass (RVL, kg·kg^−1^). Internal load was assessed by the ratings of perceived exertion (RPE) and heart rate (HR).

**Results:**

Smaller relative improvements were found for the back squat in the N group (11.5 ± 8.8%) when compared to the NH group (22.2 ± 8.2%, *P* = 0.01) and the HH group (22 ± 8.1%, *P* = 0.02). All groups showed similar RVL, HR responses and RPE across the program (*P*˃0.05). However, reduced HR recovery values, calculated as the difference between the highest HR value (HR_peak_) and the resting heart rate after a two min rest, were seen in the N and NH groups across the program (*P* $<$ 0.05).

**Conclusion:**

It seems that 8 weeks of intermittent R_T_ in hypoxic environments could maximize time-efficiency when aiming to improve strength levels in back squat without evoking higher levels of physiological stress. Performing R_T_ at hypobaric hypoxia may improve the cardiorespiratory response, which in turn could speed recovery.

## Introduction

Altitude training is widely used by coaches and athletes to complement normal training at sea level. Applied to strength, studies have shown how hypertrophy resistance training (R_T_) under moderate hypoxic conditions promote the mechanisms related to maximal strength and muscle mass development (Kurobe et al. 2015; Kon et al. 2010). The alternatives to performing R_T_ under moderate hypoxic conditions are numerous and will depend on the combination of the dosage of hypoxia (chronic *vs.* intermittent) and the type of hypoxia (hypobaric hypoxia [HH] or real altitude *vs.* normobaric hypoxia [NH]). Regarding dosage, intermittent exposure, such as “living low—training high”, seems to be more advantageous than permanent exposure. It has been observed that permanent exposure can lead to a reduction in muscle cross-sectional area and a decrease in the size of muscle fibers in humans (Narici and Kayser 1995). It can also result in increased neuromuscular fatigue during exercise by impairing neuromuscular transmission during contractions (Amann et al. 2006), and by decreasing the muscle fibers’ capacity for relaxation (Allen et al. 2008). Regarding the types of hypoxia: HH can be achieved by ascending to a real moderate altitude, while NH can be achieved by reducing the oxygen pressure in the inspired air (nitrogen dilution or oxygen filtration). For economical and logistical reasons, NH is often used as a laboratory alternative to HH, because athletes do not have to ascend to a higher altitude nor spend time acclimating. Alternative techniques can also be employed to trigger a hypoxic response in muscles. Applying pressure to the blood vessels near the skeletal muscles leads to insufficient oxygen delivery (hypoxia) to the muscle tissue (Patterson et al. 2019). Instead of decreasing atmospheric oxygen globally, hypoxia can be localized by exerting external pressure on the limbs, causing partial restriction of blood flow, a method known as blood flow restriction.

The efficiency, safety, and effectiveness of R_T_ training programs are paramount for sport conditioning. Coaches try to identify the optimal doses of the training load (product of exercise intensity and volume) since, if it is insufficient, adaptation might not occur, while excessive stress might impair performance and potentially increase the risk of injury (Halson 2014). For this reason, it becomes really important to monitor parameters of both internal (i.e., the athlete’s individual responses, such as heart rate [HR] and ratings of perceived exertion [RPE]) and external (i.e., the total volume of load lifted: sets × reps × kg) loads to quantify the training stress and hence estimate the training efficacy (McLaren et al. 2017). When evaluating the response to training stress, internal load provides a more precise and individualized measurement compared to external load. However, factors such as training status, nutrition, health, psychological status, environment (e.g., altitude, temperature) and genetics may lead to variation in their internal load when providing the same external load (Impellizzeri et al. 2018). The optimal dose of training intensity for R_T_ under hypoxic conditions may differ, as the environmental condition per se seems to be physiologically more demanding than at sea level (Rodríguez-Zamora et al. 2019). In addition, when comparing the hypoxic conditions (HH *vs.* NH), it has been reported that HH represents a more severe physiological stimulus than NH at the same theoretical altitude (Millet and Debevec 2020; Timon et al. 2022). Thus, the quantification of the training load could be distinct depending on the R_T_ environment.

Ratings of perceived exertion and HR monitoring have been widely used to verify the internal load in athletes. While RPE seems to be a valid surrogate measure of the internal load in R_T_ (McGuigan et al. 2004; Sweet et al. 2004), the use of HR monitoring as a direct indicator is scarce (Apkarian 2019; Moreira et al. 2017). Heart rate monitoring during R_T_ serves as a practical and cost-effective method to gauge the physiological response to exercise. It provides real-time insights into the cardiovascular demands of R_T_, aiding in the assessment of internal training load (de Beukelaar and Mantini 2023). Research suggests that HR can serve as a reliable indicator of exercise intensity and metabolic stress during R_T_ sessions (de Beukelaar and Mantini 2023). This approach offers a non-invasive means to monitor, and tailor individualized training programs, enhancing both safety and effectiveness. However, it is essential to consider individual variations and factors influencing HR response to ensure accurate interpretation in the context of R_T_ (Scott et al. 2017).

R_T_ involves a blend of static and dynamic contractions that elicits hemodynamic responses (Apkarian 2019; Hill and Butler 1991). When parasympathetic activity is decreased and sympathetic activity increased there is tachycardia and increased cardiac output, increased total peripheric resistance, as well as an increase in both systolic and diastolic blood pressures (Hill and Butler 1991). However, the magnitude of this response may be affected by several factors such as the exercise load, movement velocity, rest interval duration, muscle mass involved, exercise duration, age, training status and altitude exposure (Apkarian 2019; Mourot 2018).

Unfortunately, to date, there are no longitudinal investigations comparing training load markers during intermittent hypoxic R_T_ in normobaric *vs.* hypobaric hypoxia with the equivalent training in normoxia. Therefore, we aimed to compare external and internal training load markers during an 8-week R_T_ program in normoxia, intermittent normobaric hypoxia, and intermittent hypobaric hypoxia. We hypothesized that: 1) the two hypoxic groups (HH and NH) will lift higher loads across the R_T_ program compared to normoxia; and 2) real moderate altitude would lead to higher psychophysiological stress (HR and RPE) values when compared to the other two conditions.

## Methods

### Participants

Thirty-three physically trained Sport Science students familiarized with the R_T_ exercises employed volunteered to participate in the study. The mean ± SD of age, training experience, height, body mass, BMI, fat mass, and fat free mass were: 22.4 ± 3.2 years, 2.9 ± 1.8 years, 176.5 ± 6.5 cm, 73.8 ± 10.1 kg, 23.7 ± 2.8 kg∙m^−2^, 8.1 ± 4.2 kg, and 65.7 ± 6.8 kg, respectively. To be included, volunteers could have no self-reported health issues or muscle injuries and not have been exposed to more than three to four consecutive days of altitudes higher than 1500 m above sea level (MASL) for at least 2 months before the study. Participants were provided with information on the research protocol, and they provided signed informed consent. This study was approved by the local Ethics Committee (PEIBA/2018) and was conducted in accordance with the Declaration of Helsinki.

### Design

A longitudinal quasi-experimental design was employed to compare changes in the relative volume load (RVL), ratings of perceived exertion (RPE, a.u), and the heart rate (HR, bpm) response during an 8-week R_T_ program. Participants were grouped according to the following conditions: living and training in normoxia (*N*; 690 m; *n* = 10); living in normoxia and training at moderate altitude (HH; 2.320 m; *n* = 10); and living in normoxia and training at normobaric hypoxia (NH; inspired fraction of oxygen [F_i_O_2_] = 15.9%; ~ 2320 m; *n* = 13). The characteristics of each group are shown in Table 1. To see the effect of the R_T_ program on one repetition maximum (RM) in the three conditions, we compared the relative one RM values when executing back squat and bench press exercises before and after the program in normoxic conditions (PiO_2_ = 149 mmHg). The training program comprised three R_T_ sessions·week^−1^ (24 in total) at the same time of day with two additional weeks allocated for pre- and post-muscular strength and anthropometric assessments. The first session of each week (Mondays = control session) was used for the analysis. Both the N and NH groups trained at the faculty laboratory (below 700 MASL), while the HH group conducted the training sessions at the High-Performance Center in Sierra Nevada, Granada, Spain (2320 MASL). All volunteers took part in a pre-intervention 2-week conditioning-training program. To ensure the essential amino acids intake for performance during the training periods subjects were provided with a standard protein shake after each training session. To minimize the potential for instruction bias, the researchers remained the same for all the groups. Participants were instructed to abstain from intense exercise and alcohol intake, and to maintain their customary sleep and diet habits during the study.

**Table 1:** Physical characteristics and training status of participants (*n* = 33) per group

Variables	*N* (*n* = 10)	HH (*n* = 10)	NH (*n* = 13)	Interaction *P*
Age (years)	22.7 ± 3.4	22.8 ± 4.2	21.9 ± 2.2	0.7
Height (m)	175.3 ± 4.1	177.5 ± 7.4	176.5 ± 7.4	0.8
Body weight (kg)	72 ± 7.7	74 ± 13.9	75 ± 8.9	0.8
BMI (kg/m^−2^)	23 ± 2.3	23.4 ± 4.1	24 ± 1.8	0.8
Fat-free tissue mass (kg)	64 ± 4.4	66.6 ± 10.2	66.1 ± 5.3	0.7
Fat mass (kg)	8 ± 3.9	7.4 ± 5	8.9 ± 4	0.7
Training experience (years)	3.7 ± 2.3	3.7 ± 1.9	2.5 ± 1.9	0.3
Full squat 1RM (kg)	95.9 ± 14.7	80.5 ± 14.8	95.4 ± 19.2	0.1
Deadlift 1RM (kg)	104.6 ± 19.7	95.5 ± 33.8	97.7 ± 20.4	0.7
Pull down 1RM (kg)	63.8 ± 12.2	59 ± 10.5	68.9 ± 9	0.1
Barbell row 1RM (kg)	64.5 ± 12.1	58.1 ± 13.3	69.4 ± 9.5	0.1
Bench press 1RM (kg)	77.9 ± 13.5	63.9 ± 12.1	71 ± 15.8	0.1
Shoulder press 1RM (kg)	41.6 ± 6.7	37.4 ± 6.5	38.1 ± 8.3	0.4

Data are mean ± SD (*n* = 33). N = normoxia group, HH = intermittent hypobaric hypoxia group, NH = intermittent normobaric hypoxia group, 1RM: one-repetition maximum

### Methodology

#### The normobaric hypoxic condition

The NH condition was created in a normobaric tent (CAT 430, Colorado Altitude Training, USA), where oxygen-depleted air was pumped from a hypoxic generator with a semi-permeable filtration membrane (nitrogen filter technique; CAT 310, Louisville, Colorado, USA) to simulate hypoxic conditions (FiO_2_ = 15.9%, equivalent to 2320 MASL). Barometric pressure in the tent was equivalent to that of University of Granada campus (704 mmHg). Ambient O_2_ was continuously monitored by a digital controller (Handi + , Maxtec, USA) to maintain the hypoxic conditions in the tent. Immediately before and after each first R_T_ session of the week, arterial oxygen saturation (SpO_2_) was measured per duplicate using a pulse oximeter (Wristox 3100; Nonin, Plymouth, MN, USA).

#### The training session

**Fig. 1:**
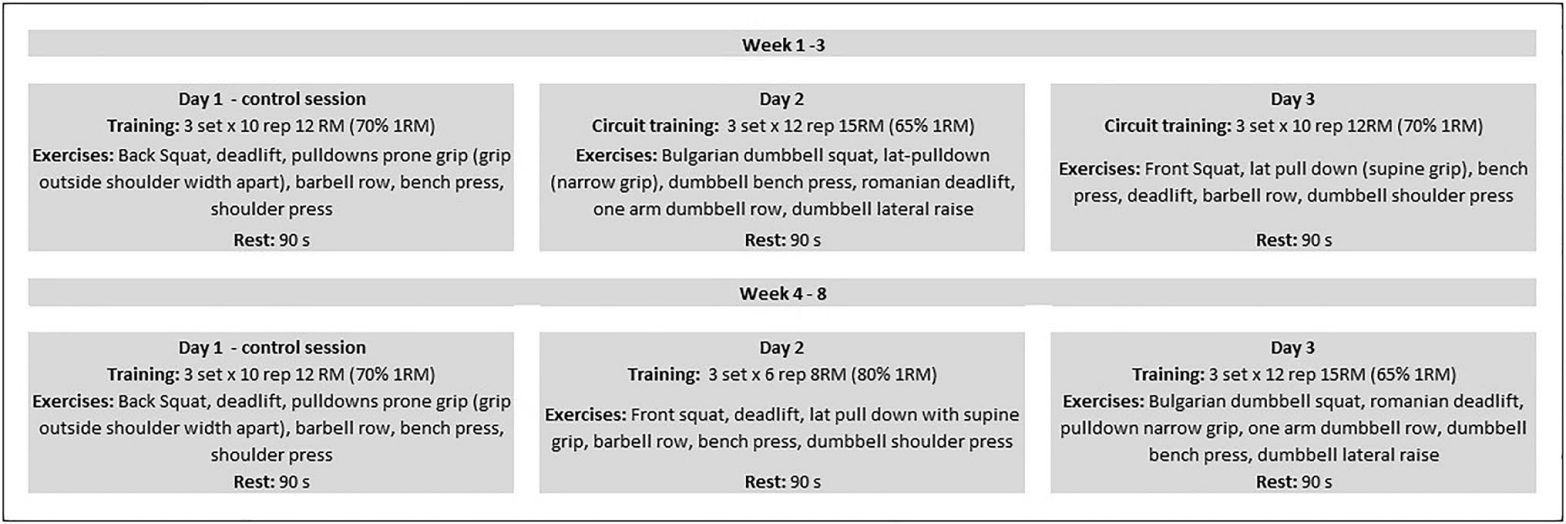
Training regime for the 8 weeks of the resistance training program

Each R_T_ session encompassed ~ 15 min of warm-up and the execution of six exercises, with three sets of six to 12 repetitions at 65–80% of 1RM with 90 s of rest between sets and exercises (Fig. 1) to promote muscle growth (Kraemer et al. 1991). During the recovery, participants were asked to sit for 2 min. The lifted load was individually adjusted in every session so the participants could perform ± 2 rep. If a participant could perform ± 3 reps, the training load was adjusted by ± 5% accordingly (Kraemer et al. 2009). All the training sessions were supervised by experts to guarantee proper technique and safe execution. Because volume load is largely related to measures of internal load and physiological stress during resistance exercise at various intensities (Genner and Weston 2014), the relative volume load (RVL, kg·kg^−1^) as well as its percentage of change (ΔRVL) were calculated for every R_T_ session and were considered for further analysis. (Relative volume load was calculated as a product of the total number of repetitions performed and the amount of mass lifted divided per body weight; ΔRVL was defined as the difference in RVL between session one and sessions two to eight, expressed in %).

#### Strength performance

One RM was estimated for each of the six main exercises of the training program (back squat, bench press, deadlift, pulldown prone grip, barbell row, and shoulder press) using a progressive trial and error procedure (Brown and Weir 2001). Three sets of three to six repetitions at increasing loads were completed before performing one set of two to three repetitions to failure. Rest periods between sets were kept to 5 min. Using the two–three RM load, one RM values were predicted using Brzycki’s equation (Brzycki 1993). After the R_T_ program, subjects repeated the same procedure for the back squat and the bench press for upper- and lower-body comparison. These exercises, back squat and bench press, were selected due to differences in training machine models between locations for the deadlift, pulldown prone grip, barbell row, and shoulder press. Relative one RM (kg·kg^−1^ of body weight) as its percentage of change (Δ%) in back squat and bench press were considered for further analysis.

#### Body composition

A segmental multi-frequency bioimpedance analyzer (Tanita BC 418 segmental, Tokyo, Japan) was used to assess weight and body composition, and height was measured by a height rod (Seca 202, Seca Ltd., Hamburg, Germany).

#### Oxygen saturation

Immediately before and after each control session, peripheral arterial oxygen saturation (SpO_2_) was measured per duplicate using a pulse oximeter fingertip sensor (Wristox 3100; Nonin, Plymouth, MN, USA).

#### Heart rate

Heart rate (HR) was continuously recorded beat-by-beat by a Polar Memory Belt HR monitor (Polar, Vantaa, Finland) and a Polar M430 watch (Polar Electro Oy, Kempele, Finland). The belt was placed on the participant’s chest ten min before the H_T_ session and removed after the recovery assessment. To minimize potential instrumentation bias, participants wore the HR monitor during the pre-intervention period. Then, data were transferred to the software Polar Flow Sync 3 (version for Windows) and exported to an Excel file. Pre-exercise HR (HR_pre_) was the average HR for the minute immediately before the start of the R_T_ session; HR_peak_ and HR_min_ were the highest and lowest HR values during the R_T_ session, while HR_mean_ was the arithmetic mean for R_T_ session. The value for heart rate recovery (HRR) was calculated as the difference between HR_peak_ and the resting heart rate after a 1 min rest (HRR_1_) and two min rest (HRR_2_) (Cole et al. 2000, 1999).

#### Ratings of perceived exertion

Rating of perceived exertion (RPE) was assessed after the R_T_ session using the Borg CR-10 scale (Noble et al. 1983). To ensure the quality of the data collected, all subjects used the CR-10 scale on three different occasions 1 week prior to starting the training program (Psycharakis 2011).

### Statistical analysis

Data were presented as means and standard deviations (± SD) unless otherwise indicated. Differences in relative and absolute RM for each exercise (back squat and bench press) were assessed using two-way mixed ANOVA, with one between-subject factor (condition: N *vs.* NH *vs.* HH) and one within-subject factor (time: pre–post-R_T_ program) and interaction terms (condition x time). Differences in Δ% were assessed using a one-way ANOVA, with one between-subject factor (condition: N *vs.* NH *vs.* HH). Differences in HR parameters, RVL, and ΔRVL were assessed using a two-way mixed ANOVA, with one between-subject factor (condition: N *vs.* NH *vs.* HH) and one within-subject factor (session: from one to eight) and interaction terms (condition x session). A three-factor mixed model ANOVA with a between-subject factor (N *vs.* NH *vs.* HH) and two within-subject factors ([session: from 1 to 8] and time [pre–post-training session]) were applied on SpO_2_. Post hoc comparisons, when justified, were performed using Bonferroni correction for multiple pairwise comparisons. Partial eta squared from the ANOVA (η^2^_p_) was interpreted using the following classification: 0.02 (small), 0.13 (medium), and 0.26 (large) (Bakeman 2005). A two-tailed Pearson product moment correlation coefficient (*r*) was used to look for relationships among RVL and HR parameters during the session. The following scales were used to interpret the magnitude of the correlations: < 0.1 trivial, 0.1–0.3 small, 0.31–0.5 moderate, 0.51–0.7 large, 0.71–0.9 very large, > 0.9 nearly perfect (Cohen 1988). Differences in RPE were determined by an ART two-way mixed ANOVA with one between-subject factor (condition: N *vs.* NH *vs.* HH) and one within-subject factor (session: from one to eight) and interaction terms (condition x session). The original RPE data first underwent ART (ARTool for Windows, version 2.1.2), and then, the ranked RPE data were analyzed with the usual ANOVA procedure (Feys 2016). The level of significance was set a priori at *P* < 0.05. Statistical analyses were conducted using SPSS Statistics for Windows (v. 22; IBM Corp., Armonk, NY).

## Results

### Strength performance

After the intervention, all three groups significantly improved both the absolute and the relative load lifted in back squat (*F* = 161.2, *P* < 0.001, η^2^_p_ = 0.8; and *F* = 149.2, *P* < 0.001, η^2^_p_ = 0.8, respectively) and bench press (*F* = 139.9, *P* < 0.001, η^2^_p_ = 0.8; and *F* = 281.8, *P* < 0.001, η^2^_p_ = 0.9, respectively, Table 2). In back squat, the absolute and the relative load lifted was significantly higher in the NH group compared to the HH group (123.8 ± 17.7 kg *vs.* 103.9 ± 15.2 kg, *P* = 0.03 and 1.6 ± 0.2 kg *vs.* 1.4 ± 0.2 kg, *P* = 0.04, respectively). However, in bench press, no differences were found among groups in absolute (*F* = 2.1, *P* = 0.1, η^2^_p_ = 0.1) nor relative values (*F* = 2.6, *P* = 0.09, η^2^_p_ = 0.1).

**Table 2:** Maximal strength (1RM) values (absolute and relative) in back squat (BS) and bench press (BP) before (pre) and after (post) the 8-week R_T_ program per group

		*N* (*n* = 10)			HH (*n* = 10)			NH (*n* = 13)		
	Exercise	Pre	Post	Δ%	Pre	Post	Δ%	Pre	Post	Δ%
Absolute load (kg)	BS	95.9 ± 14.7	111.3 ± 19.4^*^	12.9 ± 9.9^α^	80.1 ± 14.8	103.9 ± 15.2^*†^	22.5 ± 8.3	95.4 ± 19.3	123.8 ± 17.7^*^	23.2 ± 8.2
	BP	77.9 ± 13.5	86.9 ± 13.4^*^	10.5 ± 7.1	63.9 ± 12.1	73 ± 12.1^*^	12.4 ± 5.5	71 ± 15.8	83.6 ± 16.6^*^	15.4 ± 6.5
Relative load (kg·kg^−1^)	BS	1.3 ± 0.1	1.5 ± 0.2^*^	11.5 ± 8.8^β^	1.1 ± 0.2	1.4 ± 0.2^*†^	22 ± 8.1	1.3 ± 0.2	1.6 ± 0.2^*^	22.2 ± 8.2
	BP	1.1 ± 0.2	1.2 ± 0.2^*^	8.9 ± 6.1	0.9 ± 0.2	1 ± 0.2^*^	11.9 ± 5.2	0.9 ± 0.2	1.1 ± 0.2^*^	14.5 ± 5.8

Data are mean ± SD (*n* = 33) for the 8-week H_T_ program. N = normoxia group, HH = intermittent hypobaric hypoxia group, NH = intermittent normobaric hypoxia group, BS = back squat, BP = bench press, Δ% = the percentage of change (pre–post). ^*^Significant differences between pre and post (*P* ≤ 0.005). Significant differences (*P* < 0.05) among groups were noted: ^α^N *vs.* NH, ^†^HH *vs.* NH, and ^β^N *vs.* HH and NH

Regarding changes, lower Δ_%_ in the absolute load lifted were found for the back squat in N when compared to the NH group (12.9 ± 9.9% *vs.* 23.2 ± 8.2%, *F* = 4.5, *P* = 0.03, η^2^_p_ = 0.2, Table 2) with no significant differences in bench press among groups (*F* = 1.7, *P* = 0.2, η^2^_p_ = 0.1). Similarly, lower Δ_%_ in the relative load lifted were found for the back squat in the N group (11.5 ± 8.8%) when compared to the NH group (22.2 ± 8.2%, *P* = 0.01) and the HH group (22 ± 8.1%, *P* = 0.02). However, no significant differences were found for this parameter in bench press among groups (*F* = 2.8, *P* = 0.08, η^2^_p_ = 0.2).

### The displaced external load

**Fig. 2:**
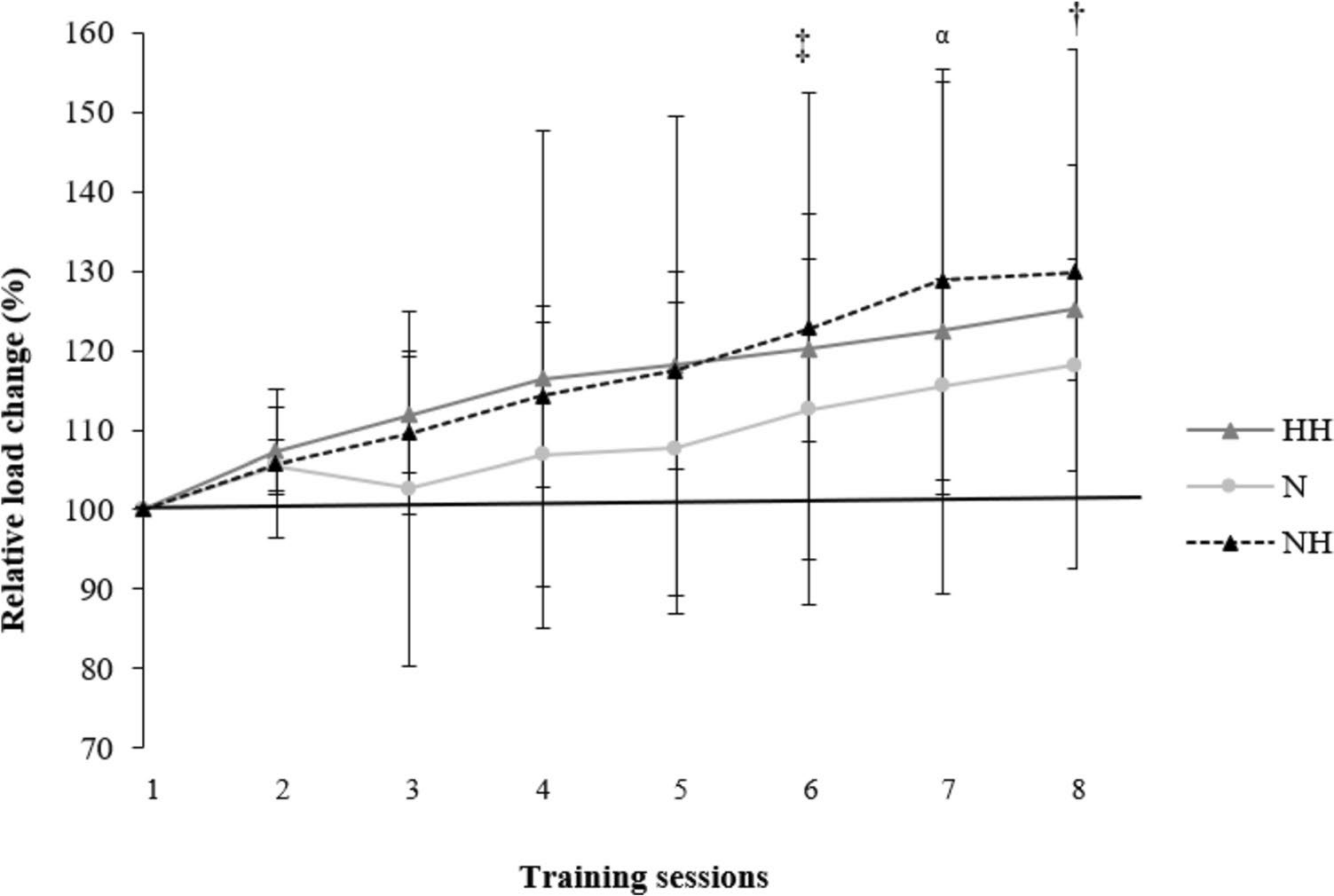
Change in the training load for the 8 weeks of the resistance training program

The RVL distribution across the R_T_ program was similar in all three groups (*F* = 0.7, *P* = 0.7, η^2^_p_ = 0.05), with a significant main effect for “session” (*F* = 15.4, *P* < 0.001, η^2^_p_ = 0.4; Table 3). Similar ΔRVL across 8 weeks were found for all three groups (*F* = 0.6, *P* = 0.8, η^2^_p_ = 0.04), with a significant main effect for “session” (*F* = 12.4, *P* < 0.001, η^2^_p_ = 0.3; Fig. 2). At the end of the R_T_ program, mean ΔRVL values were *N*: 20.4 ± 14.6% *vs.* HH: 25 ± 12.3% *vs.* NH: 31.5 ± 15.4%; (*F* = 0.9, *P* = 0.4, η^2^_p_ = 0.05) in their respective conditions.

**Table 3:** Relative volume of load lifted (RVL) and mean values of heart rate (HR) per session

	Session							
Parameter	1	2	3	4	5	6	7	8
RVL (kg)	112.8 ± 17.7	119.5 ± 18.2	121.1 ± 23.1	125.4 ± 21.5	127.1 ± 22.5^¥^	132.2 ± 22.5^δ^	136.7 ± 25.3^**^	139 ± 21.8^§^
HR_pre_ (bpm)	65.6 ± 6.8^*^	78.1 ± 14	75.4 ± 12.9	74.2 ± 13.9	75.7 ± 10.6	75.9 ± 12.5	74.8 ± 11.8	79.8 ± 13.4
HR_mean_ (bpm)	144.2 ± 14.2	145.4 ± 15.4	144.4 ± 12.5	145.3 ± 15.8	144.8 ± 15.7	144.5 ± 14.6	145 ± 12.5	148.4 ± 12.9
HR_min_ (bpm)	102.4 ± 17.5	101.9 ± 20.6	102.2 ± 16.4	103.1 ± 17.9	107.4 ± 18.7	103.8 ± 19.3	104.7 ± 17.1	113.3 ± 16.8^†^
HR_peak_ (bpm)	174.6 ± 12.2	174.7 ± 11.3	174.5 ± 11.4	175.2 ± 12.4	173.4 ± 13.2	174.1 ± 11.9	174.9 ± 10.6	175.4 ± 11.5
HRR_1_ (beats)	32.3 ± 11.3^α^	35.4 ± 14.4^α^	28.8 ± 11.2^β^	42.6 ± 15.4	45.7 ± 13.7	45.9 ± 14	47.4 ± 14.1	44.3 ± 11.8
HRR_2_ (beats)	59.2 ± 14.4^Ω^		49.7 ± 12.3	49 ± 13.2	51 ± 9.2	49.2 ± 11.5	52.7 ± 11.2	57.1 ± 9.2^Ω^

Data are mean ± SD (*n* = 33) for the 8-week R_T_ program. *RVL* relative volume of load lifted calculated as a product of the total number of repetitions performed and the amount of mass lifted divided per body weight, *HR*_*pre*_ average HR for the minute immediately before the session, *HR*_*mean*_ the arithmetic mean for the session HR_min_ and *HR*_*peak*_ the lowest and highest HR value during the session, *HRR*_*1*_ the difference between the HR_peak_ and the resting heart rate after a 1 min rest, *HRR*_*2*_ the difference between the HR_peak_ and the resting heart rate after a 2 min rest. Significant differences (*P* < 0.05) among sessions were noted:

^¥^*vs.* 1st; ^δ^*vs.* 1st, 4th and 5th; ^**^vs. 1st, 2nd, 3rd and 4th; ^§^*vs.* 1st, 2nd, 3rd, 4th, 5th and 6th; ^*^vs. 2nd, 3rd, 5th, 6th, 7th, and 8th; ^†^*vs.* 1st, 2nd, 3rd, 4th, 6th, and 7th; ^α^*vs*. 5th, 6th, 7th, and 8th; ^β^*vs.* 4th, 5th, 6th, 7th, and 8th; and ^Ω^*vs*. 3rd, 4th and 6th

### Internal load markers

The average SpO_2_ values were significantly lower in HH and NH compared to N, both before (HH: 93.8 ± 1.5 and NH: 94 ± 2.2 *vs.* *N*: 97.7 ± 1.3%; *F* = 56.5, *P* < 0.001, η^2^_p_ = 0.8) and after the program (HH: 93.5 ± 1.3 and NH: 92.5 ± 2.7 *vs.* *N*: 96.3 ± 1.1%; *F* = 113.7, *P* < 0.001, η^2^_p_ = 0.9). Significant differences between pre- and post-session SpO_2_ values were found in NH (*F* = 19.2, *P* < 0.001, η^2^_p_ = 0.2) and N (*F* = 44.9, *P* < 0.001, η^2^_p_ = 0.4), with lower values observed after the session.

The pattern of HR response was similar in most of the HR parameters for all three groups. However, a significant main effect for “session” was found in HR_pre_ (*F* = 8.6, *P* < 0.001, η^2^_p_ = 0.2) and HR_min_ (*F* = 5, *P* < 0.001, η^2^_p_ = 0.1; Table 3).

There were no significant differences among groups in HRR_1_ (*N*: 39.2 ± 11.1 beats, HH: 36.1 ± 17.3 beats, NH: 44.4 ± 14 beats, *F* = 1.4, *P* = 0.2, η^2^_p_ = 0.8). However, there was a main effect for “session” (*F* = 24.6, *P* < 0.001, η^2^_p_ = 0.5; Table 3). A significant condition x session interaction effect was found in HRR_2_ (*N*: 52.8 ± 12.1 beats, HH: 53.9 ± 13.2 beats, NH: 51.4 ± 11.4 beats, *F* = 2.8, *P* = 0.006, η^2^_p_ = 0.2), with the *N* group showing reduced values at sessions three (*P* < 0.001), five (*P* = 0.01), and six (*P* = 0.003), and the NH group showing reduced values at sessions four (*P* = 0.01), 6 (*P* = 0.02), and seven (*P* = 0.04, Table 4).

**Table 4:** Heart rate recovery at 2 min (HRR_2_) per group and per session

Parameter	Sessions							
	Group	1	3	4	5	6	7	8
HRR_2_ (beats)	N	66.4 ± 6.4^*^	43.9 ± 6.8	51.1 ± 17.3	46.4 ± 9.4	48.1 ± 7.8	53.8 ± 10.4	59.8 ± 7.2^†^
	HH	52.6 ± 16	52.3 ± 17.4	50.5 ± 14.5	53.8 ± 10	54.9 ± 11.8	55.4 ± 11.9	57.6 ± 12.3
	NH	58.7 ± 15.7^‡^	52.1 ± 10	46.2 ± 8.7	52.4 ± 7.6	45.7 ± 12.6	49.9 ± 11.5	54.7 ± 7.8^§^

Data are mean ± SD (*n* = 33) for the 8-week R_T_ program. HRR_2_: the difference between the HR_peak_ and the resting heart rate after a 2 min rest. Significant differences (*P* < 0.05) among sessions were noted: ^*^vs. 3rd, 5th and 6th; ^†^*vs.* 3rd and 6th; ^‡^*vs.* 4th, 6th and 7th; ^§^*vs.* 4th and 6th

No significant differences in RPE were found among groups (*F* = 0.3,* P* = 0.7) nor between sessions (*F* = 0.8,* P* = 0.5). Even a significant condition x session interaction effect was found (*F* = 4.3, *P* = 0.026, η^2^_p_ = 0.3), pairwise comparisons showed no significant differences (*P*˃0.05).

## Discussion

This is the first study to monitor and compare both external and internal load markers during an 8-week R_T_ program in three different environments: normoxia, intermittent hypobaric hypoxia, and intermittent normobaric hypoxia. Our research led to three principal findings: (1) the RVL dynamics were similar in all three groups across the 8-week program, with the hypoxic groups achieving larger improvements in relative 1RM back squat; (2) the indices of physiological stress (HR and RPE) during the R_T_ program seemed to be similar in all three groups; and (3) the exposure to hypobaric hypoxia seems to speed recovery during R_T_.

In our study, all three groups showed similar RVL dynamics during the R_T_ program (Table 3), with an increased RVL at the end (Fig. 2). In agreement with recent studies (Inness et al. 2016; Guardado et al. 2020), the HH and NH groups showed higher values of relative 1RM in back squat compared to the *N* group after the program. It is well known that R_T_ in intermittent hypoxic conditions produces some physiological adaptations resulting in higher levels of both muscle hypertrophy and maximal strength compared to the same R_T_ performed in normoxia (Kurobe et al. 2015; Kon et al. 2010). So, why did the relative one RM for bench press not improve? The benefit of R_T_ under hypoxic conditions is based on the large accumulation of metabolic byproducts due to hypoxia, such as blood lactate, protons (H^+^), calcium, and inorganic phosphorus, among others, derived from the increase in anaerobic metabolism to compensate the loss of oxygen availability (Kon et al. 2012; Kurobe et al. 2015; Schoenfeld 2013). In the back squat, where more muscle mass is at work, a larger accumulation of metabolic waste byproducts occurs with the concomitant larger improvement in the strength levels. In addition, the observed distinctions in back squat performance could also be attributed to the program's design, particularly the inclusion of two lower-body push exercises *vs.* one upper-body push exercise as illustrated in Fig. 1.

Concerning the HR, all three groups showed similar HR responses across the R_T_ program. This is in line with Scott et al. (2017), who reported similar HR responses between a normoxic group (FiO_2_ = 21%) and a hypoxic group (FiO_2_ = 16%) during a R_T_ session (Scott et al. 2017). It is well known that there is a linear relationship between SpO_2_ and HR. While at sea level, SpO_2_ hovers around 98–100%; above 3100 MASL (FiO_2_ = 14%), SpO_2_ drops to 80% due to the lower PaO_2_, requiring the heart to increase the HR to maintain appropriate oxygen delivery to tissues (Cardiology 2012). In our study, on average, the difference in SpO_2_ between the hypoxic groups and the N group both before (-3.8%) and after the session (-3.3%) would not have been enough to significantly modify the HR pattern. Another possible explanation could rely on the intrasubject variation. Expressed in bpm, the day-to-day variations in submaximal HR could be estimated at around five–eight bpm (Lamberts et al. 2004). This means that the expected difference between normoxia and hypoxia in terms of the HR pattern at moderate altitude (for instance, our 2320 MASL) could be hidden by the spontaneous fluctuations of HR for a given training load. Furthermore, it is well known that other factors such as endurance training experience and genetics could have also affected the HR response of our participants (Mollard et al. 2007; Masschelein et al. 2015).

In the present study, the HH group showed larger HRR_2_ values during a few sessions of the R_T_ program (Table 4) showing faster recovery. According to the literature, higher levels of HRR_2_ are indicative of a more efficient cardiovascular system, suggesting that the heart can rapidly return to baseline levels after exertion, which is generally associated with better cardiovascular fitness and health (Cole et al. 2000). HRR_2_ is influenced by the gradual decrease in sympathetic activity and the removal of metabolites (such as epinephrine, lactate, and H^+^) from the bloodstream, both of which are consequences of engaging in intense exercise (Imai et al. 1994). Terrestrial altitude in comparison to simulated leads to grater hypocapnia and blood alkalosis for the equivalent “altitude” due to the barometric pressure reduction (Savourey et al. 2003). In this sense, performing 2 months of R_T_ at moderate hypobaric hypoxia may produce cardiorespiratory adaptations which in turn would speed recovery (Álvarez-Herms et al. 2012; Savourey et al. 2003). Our findings are consistent with those of Bhattarai et al. (2018), who observed comparable basal HR but significantly faster recovery of HR after step test in highlanders (~ 3000 MASL) *vs.* lowlanders (Bhattarai et al. 2018) and with those of Álvarez-Herms et al. (2012), who found better HRR_2_ values after 4 weeks R_T_ in a hypobaric chamber compared to normoxia (Álvarez-Herms et al. 2012).

Regarding RPE, the Borg scale CR-10 has been proposed as a surrogate measure for the internal load during R_T_ (Sweet et al. 2004; McGuigan et al. 2004). The lack of differences in RPE among groups could be explained by the same intensity applied (~ 70% 1RM) in all three conditions (Gearhart et al. 2002) and the dosage of hypoxia. Progressive arterial hypoxemia and increases in ventilation have been identified as the primary cues for determining RPE in moderate (FiO_2_ = 15.2%) and severe hypoxic (FiO_2_ = 11.4%) environments (Jeffries et al. 2019). It seems that in our study, the averaged SpO_2_ achieved in the hypoxic groups (HH: 93.5 ± 1.3 and NH: 92.5 ± 2.7) was not enough to trigger a decline in the perceptual responses. Attenuated RPE after hypoxic training has been described (Brocherie et al. 2017), suggesting an improved tolerance or acclimatization to hypoxia after only one session. In contrast, R_T_ in hypoxia has been perceived as tougher (Inness et al. 2016; Rodríguez-Zamora et al. 2019) or without effect (Scott et al. 2015). These discrepancies may be attributed to the Borg scale used (6–20 *vs.* CR-10) and the athletic experience (Noble and Robertson 1996; Barroso et al. 2014). Future work should explore which parameters can explain the perceived exertion in R_T_ under hypoxic conditions and to determine which internal load markers could potentially help the most to provide information about the athlete’s physiological stress in hypoxic environments.

Certain issues and limitations regarding the design, methodology, and overall validity of this study need to be considered. To include as many participants as possible in each group, it was decided to make the groups according to the subjects’ availability. Hence, there could have been some intrinsic characteristics of the groups (differences in motivation, personality, stress, etc.) that could have influenced the results of the study. In addition, the exact timing for parasympathetic reactivation post-exercise is likely to be highly impacted by an individual’s health and training status, and thus it is likely to vary from person to person. However, the within-subject design would have helped to minimize any potential variations attributed to that fact. In terms of methodology, the inclusion of markers for muscle activation and blood/urine samples could have offered valuable insights into the physiological responses to our intervention. However, a conscious decision was made to streamline our study design, prioritizing feasibility and practicality to address our primary research question. Studies with a larger sample size are recommended. Finally, due to technical problems with the HR devices, HRR_2_ for session two was not calculated.

## Conclusions

The results of this study suggest that 8 weeks of intermittent R_T_ in a hypoxic environment could help to maximize time-efficiency when aiming to improve strength levels in back squat. However, further research is needed. It seems that athletes may lift similar loads (%RM) compared to what can be lifted in normoxia without evoking higher levels of physiological stress. In addition, performing intermittent R_T_ at hypobaric hypoxia may improve the cardiorespiratory response, which in turn could speed recovery. This is of considerable relevance for coaches because it highlights the importance of not only applying the appropriate training loads but also selecting the proper muscular environment during R_T_.

## Data Availability

The raw data supporting the conclusions of this manuscript will be made available by the authors, without undue reservation, to any qualified researcher.

## Abbreviations

ANOVA: Analysis of variance
BMI: Body mass index
F_i_O_2_: Inspired fraction of oxygen
HH: Hypobaric hypoxia
HR: Heart rate
HR_pre_: Average HR for the minute immediately before the start of the R_T_ session
HR_min_: Lowest HR value during the R_T_ session
HR_peak_: Highest HR value during the R_T_ session
HR_mean_: Arithmetic HR mean for R_T_ session
HRR: Heart rate recovery
HRR_1_: HRR calculated as the difference between HR_peak_ and the resting heart rate after a 1 min rest
HRR_2_: HRR calculated as the difference between HR_peak_ and the resting heart rate after a 2 min rest
MASL: Meters above sea level
NH: Normobaric hypoxia
N: Normoxia
PaO_2_: Oxygen partial pressure
RM: Repetition maximum
Δ%: Repetition maximum percentage of change
RVL: Relative volume load
ΔRVL: Relative volume load percentage of change
R_T_: Resistance training
RPE: Rating of perceived exertion
SpO_2_: Arterial oxygen saturation
SD: Standard deviation
